# Is the future of peer review automated?

**DOI:** 10.1186/s13104-022-06080-6

**Published:** 2022-06-11

**Authors:** Robert Schulz, Adrian Barnett, René Bernard, Nicholas J. L. Brown, Jennifer A. Byrne, Peter Eckmann, Małgorzata A. Gazda, Halil Kilicoglu, Eric M. Prager, Maia Salholz-Hillel, Gerben ter Riet, Timothy Vines, Colby J. Vorland, Han Zhuang, Anita Bandrowski, Tracey L. Weissgerber

**Affiliations:** 1 BIH QUEST Center for Responsible Research, Berlin Institute of Health at Charité Universitätsmedizin Berlin, Berlin, Germany; 2 Australian Centre for Health Services Innovation and Centre for Healthcare Transformation, School of Public Health & Social Work, Queensland University of Technology, Brisbane, QLD Australia; 3 NeuroCure Cluster of Excellence, Charité Universitätsmedizin Berlin, Berlin, Germany; 4 Department of Psychology, Linnaeus University, Växjö, Sweden; 5 Faculty of Medicine and Health, New South Wales Health Pathology, The University of Sydney, New South Wales, Australia; 6 Department of Neuroscience, University of California, San Diego, La Jolla, CA USA; 7 UMR 3525, Institut Pasteur, Université de Paris, CNRS, INSERM UA12, Comparative Functional Genomics group, Paris, France; 8 School of Information Sciences, University of Illinois Urbana-Champaign, Champaign, IL USA; 9 Translational Research and Development, Cohen Veterans Bioscience, New York, NY USA; 10 Faculty of Health, Center of Expertise Urban Vitality, Amsterdam University of Applied Science, Amsterdam, The Netherlands; 11 DataSeer Research Data Services Ltd, Vancouver, BC Canada; 12 Indiana University School of Public Health-Bloomington, Bloomington, IN USA; 13 School of Information Studies, Syracuse University, Syracuse, NY USA

**Keywords:** Rigor, Reproducibility, Transparency, Automated screening, Peer review

## Abstract

The rising rate of preprints and publications, combined with persistent inadequate reporting practices and problems with study design and execution, have strained the traditional peer review system. Automated screening tools could potentially enhance peer review by helping authors, journal editors, and reviewers to identify beneficial practices and common problems in preprints or submitted manuscripts. Tools can screen many papers quickly, and may be particularly helpful in assessing compliance with journal policies and with straightforward items in reporting guidelines. However, existing tools cannot understand or interpret the paper in the context of the scientific literature. Tools cannot yet determine whether the methods used are suitable to answer the research question, or whether the data support the authors’ conclusions. Editors and peer reviewers are essential for assessing journal fit and the overall quality of a paper, including the experimental design, the soundness of the study’s conclusions, potential impact and innovation. Automated screening tools cannot replace peer review, but may aid authors, reviewers, and editors in improving scientific papers. Strategies for responsible use of automated tools in peer review may include setting performance criteria for tools, transparently reporting tool performance and use, and training users to interpret reports.

## Introduction

Peer review is a cornerstone of scientific publishing that, ideally, provides high quality assessments on large numbers of submitted manuscripts. Rising publication rates have increasingly strained this system. While many papers benefit from peer review, problematic papers are still published [1]. This may include papers with fundamental flaws in design, analysis or inference, or fraudulent papers. Correcting errors after publication is extremely burdensome [2, 3]; hence, focusing on prevention may be more efficient. Inadequate reporting is also common in published studies [4–6], making it difficult for reviewers to evaluate manuscripts. Published papers are routinely missing information needed to assess the risk of bias. Statistical errors are also common [7, 8]. Evidence that peer review substantially improves reporting, or catches errors or questionable research practices, is limited [9, 10]. The lack of a comprehensive reviewer training system may contribute to problems with peer review [11].

Automated screening in academic publishing is not new, and may offer a unique opportunity to improve scientific papers. Publishers have been using automated tools to detect plagiarism for more than a decade [12]. Journals could potentially use screening tools to improve reporting before sending papers to reviewers, or enhance peer review by drawing reviewers’ attention to opportunities for improvement. The growing adoption of preprints offers another opportunity to use automated tools to help authors to improve their papers [13]. While preprints allow scientists to receive feedback before publishing their work in a journal, comments on preprints are uncommon [14]. Automated tools could help to fill this gap. Some publishers are experimenting with using automated tools to check for factors such as statistical reporting errors [15], ethics statements, blinding, randomization and sample size calculations [16].

Our experience suggests that automated screening is most powerful when many tools are applied simultaneously to assess various aspects of reporting. The ScreenIT pipeline, which includes a growing set of automated tools, has been used to post public reports on more than 23,000 bioRxiv and medRxiv COVID-19 preprints [17]. While this approach was adopted to support authors and readers in assessing the flood of COVID-19 preprints, it demonstrates the feasibility and potential of widespread automated screening. Table 1 provides a brief overview of some tools that have been used to screen preprints or papers. Given these developments, it is important to consider the strengths and limitations of automated screening and how one might responsibly integrate these tools into the editorial process.

**Table 1:** Examples of automated tools used to screen preprints, submitted papers or publications

Tool	Screening topics and rationale
Sciscore [18]	Many factors, including: RRIDs: Unique persistent identifiers that allow readers to determine exactly what resource (e.g., cell line, antibody, model organism, software) was used Ethics & consent statements: Required for legal compliance Blinding & randomization: The failure to blind or randomize experiments is associated with overestimated effect sizes Sample size calculations: Provide information about whether the study was designed and powered to detect an effect of an expected size Sex/gender: Effects may differ according to sex or gender
ODDPub [19]	Open data, open code: Open data and open code make it easier to reproduce analyses, identify potential errors, and re-use data
Limitation-recognizer [20]	Author-acknowledged limitations: Every study has limitations. Acknowledging limitations provides essential context that allows readers to interpret the study results
Barzooka [21]	Bar graphs of continuous data: Many datasets can lead to the same bar graph and the actual data may suggest different conclusions from the summary statistics alone. These graphs should be replaced with dot plots, box plots or violin plots
Jetfighter [22]	Rainbow color maps: Rainbow color maps are not colorblind accessible, and create visual artifacts for readers with normal color vision
Trial registration number screener	Clinical trial registration numbers: Clinical trials must be registered in an International Clinical Trials Registry Platform registry, and this number must be reported in publications. This makes it easier to detect practices like outcome switching
Statcheck [7]	Misreported p-values: p-values that do not match the reported test statistic and degrees of freedom are common and can sometimes alter study conclusions
Scite reference check	Citation of retracted papers, or papers with corrections or errata: Checking cited papers for editorial notices can help to identify potentially problematic citations
Seek and blastn (semi-automated) [23]	Confirms that nucleotide sequences were correctly identified: Incorrect identification or use of nucleotide sequences makes it difficult to interpret or reproduce study results. Results from this tool require confirmation from an expert reviewer

## Main text

### How can automated screening help peer review?

Peer review includes three areas of assessment: journal fit, research and reporting quality, and compliance. The “fit” assessment considers whether the manuscript aligns with the journal’s aims and scope and is typically performed by journal editors or administrators [24]. Fit may also include basic assessments, such as confirming that the submission is a legitimate scientific paper that falls into one of the journal’s accepted article types. The research and reporting quality assessment examines many factors, including scientific rigor, novelty, anticipated impact, significance to the field, relevance to medical practitioners, the wider scientific community and society, and the quality of writing and data presentation. This broad assessment is typically performed by reviewers, although editors may also contribute. Compliance assessment determines whether the article complies with relevant policies. This includes ethical standards (e.g., plagiarism, consent, or ethical approval statements), funder requirements (e.g., grant numbers, clinical trial registrations), and journal requirements (e.g., compliance with formatting guidelines, reporting guidelines or open data policies). The journal editorial office may assess aspects of compliance, although reviewers may also comment on adherence to reporting guidelines or other compliance elements that impact research and reporting quality. The compliance and research and reporting quality assessments provide authors with valuable feedback, while all three assessments help editors to decide which papers to publish.

We believe that in their current form, automated tools have the most potential to aid in assessing compliance. This may also include some routine aspects of the research and reporting quality assessment (e.g., compliance with elements of reporting guidelines, such as CONSORT [25], PRISMA [26], or ARRIVE [27]). The broader research quality and journal fit assessments are best left to expert human reviewers and editors. While limited in scope, using automated tools to aid in assessing compliance and basic reporting quality items would fill an important gap. Editorial offices often lack the expertise and capacity to check all compliance criteria. Many “best practice” criteria are routinely neglected by reviewers and editors. These include criteria such as following reporting standards, or transparently presenting statistical results [28].

### Strengths and limitations of automated screening

Automated screening tools may be able to address several limitations of peer review [24]. Traditional peer review often fails to address widely accepted, but suboptimal, research practices and guideline details. Examples include incomplete reporting of criteria to assess risk of bias [6], ambiguous or incorrect citations [29], lack of open data or code [30], incorrect statistical calculations [31], and underreporting of ethics [32], sex as a biological variable [33], and limitations statements [34]. Whereas traditional peer review requires time and effort [35], tools can quickly screen many papers and provide individualized feedback on some of the items included in transparency and reporting guidelines. Automated screening may also raise awareness of the existence of guidelines and the need for better practices. In addition to detecting potential problems or missing information, tools can also detect beneficial practices (e.g. open data, open code). Tools can be adapted to assess different types of studies, such as in vitro, preclinical or clinical research, or different study designs.

Despite these advantages, automated tools have important limitations [17]. Tools make mistakes. They cannot always determine whether an item is relevant to a given paper, especially when reporting is poor. Furthermore, tools that assess reporting quality may not capture information that reflects the methodological quality of the experiment. Automated screening tools typically use algorithms or machine learning to recognize patterns, with varying levels of sophistication. Existing tools are not capable of understanding or interpreting the research in the context of the scientific literature. They cannot determine whether the methods used are suitable to answer the research question, or whether the data support the authors’ conclusions. A deeper understanding is essential for assessing innovation, impact, and some elements of scientific rigor. Notably, many of these limitations may also apply to human reviewers, especially those who are not trained in peer review or are reviewing papers outside their area of expertise.

### Considerations for responsible use of automated screening

Within the editorial process, potential users of automated tool reports include authors, journal editors, administrative staff, and reviewers. Introducing tools into the editorial process requires careful consideration and pilot testing. Reports should be interpreted by a knowledgeable reader and could be targeted across phases and to different stakeholders, such as journal editors and peer reviewers. Simply introducing reports into a system where many peer reviewers receive minimal training in manuscript review may have unintended consequences. Some reviewers might uncritically rely on the reports, rather than using the reports as supplemental information and focusing on impact, innovation, and other factors that the existing tools cannot reliably assess [36]. Authors and reviewers who are not familiar with the reports, or who regularly use suboptimal practices identified by tools, may not understand why the items mentioned in reports are important or how to implement better practices. All users should also be aware that tools make mistakes. Tool performance, as measured by F1 scores, sensitivity and specificity, should be transparently reported, along with known performance issues to aid all users in gauging the effectiveness of the tools. F1 scores are calculated as the harmonic means of precision and recall.

Integrating automated screening into the editorial process also requires technical solutions. Adding new tools to manuscript submission systems is time consuming and can be expensive. Sometimes publishers expect tool developers to cover these costs, which far exceed project budgets for open source tool developers. Systems to quickly and inexpensively integrate tools into manuscript submission platforms are urgently needed.

There are also many opportunities to expand and improve the tools themselves. ScreenIT shows that integrating tools into a combined pipeline allows us to screen for more features, including criteria that are relevant to different study designs or disciplines. Furthermore, ScreenIT includes several instances where different tools screen for similar items. These include features like open data and open code, clinical trial registrations, the use of problematic cell lines, and attrition. Even in these cases, our experience indicates that combining reports from multiple tools gives a more complete picture than using a single tool. Different tools may screen different parts of the manuscript, detect different criteria, or be optimized for different types of papers. Publishers will want to select the subset of tools that meets their needs, or adapt the tools to suit their reporting requirements. Automated tools could also be developed for other applications, such as trial registries and funding applications.

Several other factors should be considered to ensure that automated screening tools meet the scientific community’s needs. Research should systematically assess factors that one could examine with automated screening, and identify those that have the most impact on the interpretation of study results. This would guide tool developers in determining what types of tools are most urgently needed. The level of reporting that a tool detects is also important. A tool to detect blinding, for example, could be designed to determine whether any statement about blinding is present, whether blinding was used at any phase of the study, or whether individual stakeholder groups were blinded (e.g., patients, caregivers, outcome assessors, or data analysts). Tools that detect any statement may be most useful for items that are rarely addressed, whereas tools that assess nuanced reporting are better for commonly reported items.

Finally, we need to consider the user experience and needs of the scientific community. Reports should be carefully designed, with feedback from researchers and publishers, and combined with educational materials to provide authors with clear guidance about how to improve their paper. The scientific community needs to identify the most responsible way to share reports. At what phase of peer review should reports be shared with editors, peer reviewers, and authors? When screening preprints, should reports be shared only with the authors, reviewers, and editors, or should reports be publically available to readers? We also need standards for transparently reporting tool performance and limitations, and determining how these criteria should factor into the reporting and interpretation of tool results. If automated screening becomes widespread, publishers and toolmakers may need to protect against gaming.

## Outlook

Editors and peer reviewers are essential for assessing journal fit and research and reporting quality, including scientific rigor, the soundness of the study’s conclusions, potential impact, and innovation. Automated screening tools may play a valuable supporting role in assessing compliance and some elements of research and reporting quality, such as compliance with reporting guidelines. Automated screening may also be useful in systematically raising awareness about the problems with widely accepted, suboptimal practices that might be overlooked in peer review. While the future of peer review may include reports from automated tools, knowledgeable reviewers should use these reports responsibly. Future work should enhance existing tools, simplify integration of tools into editorial systems, and train reviewers, editors and authors to use tool reports to improve papers. If successful, automated tools could reduce poor reporting and educate researchers about reporting best practices.

## Data Availability

No data or materials were generated during preparation of this commentary.

## Abbreviations

APA: American Psychological Association
RRID: Research resource identifier
